# Abstract of the Proceedings of the Buffalo Medical Association, May 7th, 1878

**Published:** 1878-06

**Authors:** Joseph Fowler

**Affiliations:** Secretary


ART. IV.—*Abstract of the Proceedings of the Buffalo Medical Association.* May 7, 1878.

Members in attendance: Doctors Lothrop, Gay, Cronyn, Gould, Mynter, Brecht, Howe, Wyckoff, Rochester, Bartlett, S. S. Greene, Ring and Fowler, and by invitation Dr. Harting, who on motion was invited to participate in the proceedings of the Association until such time as was necessary to complete his membership.

Dr. C. C. F. Gay, the essayist for the evening, presented "Clinical Notes and Comments concerning the Surgery of the Hip, with a Résumé of Methods of Admeasurement." The main feature of the paper was to point out the uncertainty of physical signs in the diagnoses of injuries to the hip, and cases were cited to show that some of the most eminent surgeons had been led into error and forced to an acknowledgment by subsequent *post mortem* appearances. The paper concluded with a few remarks upon the various methods employed to detect deformity by measurement, the reader being of the opinion that no one method could be considered perfectly reliable, and that it is well to be familiar with them all and avail ourselves of the advantages each possesses.

In the discussion which followed, Dr. Wyckoff said a sensible method of detecting shortening was to require the individual to stand erect, and pass a tape line from the seventh cervicle vertebra to some fixed point of the foot, then elevate the extremity which is the shorter by placing a wedge under the foot until the distances from the points of measurement on either side are equal. The thickness of the wedge would indicate the degree of shortening.

Dr. Greene thought the method rather a tedious one, but could see no reason why it should not be accurate. It would be a difficult matter for some patients to stand on their feet. He believed the best way was to put the patient on his back with his hips level and measure from the anterior superior spinous process to the internal malleolus. If the distances between these points are not uniform in healthy persons who have never suffered from disease of the hip or extremity, as appears to be proven by the table of measurement made upon such individuals referred to in Dr. Gay's paper, then he doubted the accuracy of this method.

Dr. Mynter did not think that the method mentioned by Dr. Wyckoff a reliable one, as the person might incline to one side and the difference in measurement would simply indicate lateral curvature of the spine. He believed the most accurate way was to place the patient on his back and carry a bar across the hips at right angles with the median line, then measure from this bar on either side to the internal malleolus.

Dr. Cronyn understood that the principle object of Dr. Gay's paper was to call our attention to certain means employed to ascertain whether the hip had been injured in one way or another, and quoted cases to prove that the most skillful had been in error. The head of the bone may be displaced from its socket without evidence of shortening in one case, and in another the limb may be considerably shorter, and no one method of measurement can be relied upon in all these cases, but all must be tried. The majority of surgeons in London use Bryant's angular method. He had removed the head of the humerus and found no difference afterwards in its length as compared with the other arm, thus proving that a similar condition of the hip might exist without evidence of shortening.

Under the head of Voluntary Communications Dr, Howe presented a case illustrating the result of operative treatment in divergent strabismus. The patient, K. B., was a girl of fifteen, whose eyes were healthy up to seven years ago. At that time there was developed in the left one a severe attack of purulent conjunctivitis. A leucoma, or nebula remained which covered the pupil, and the entire internal and inferior quadrant of the cornea, and there followed in this case, as in many similar ones, a divergent strabismus of the blinded eye. Moreover, the deviation from the normal position was so great that when the right eye was directed at an object immediately in front, the left had part of its cornea covered by the external angle of the lids. The resulting deformity together with the bleared appearance presented, could be removed only by first hiding the white spot of the cicatrix, and secondly returning the eye to its natural position. The former indication was fulfilled by smearing the corner with india ink and tattooing it in, by means of a fine needle. The latter was accomplished as follows: The internal rectus muscle, being severed from the sclerotic, a stitch was passed through its extremity and it was brought forward, to be attached close to the margin of the cornea. The external rectus was also divided, but a fragment was left next to the globe, to which a thread several inches long was made fast. This thread was then drawn across the nose, and being retained there by means of a strip of plaster, the eye was thus forcibly rotated inwards. This position was retained for three days, the internal rectus having been made to advance, and the external rectus to recede from their former position. As a result, the artificial coloring of the cornea could only be detected upon examination, and the divergence reduced to such a degree that although present it was not markedly apparent.

Dr. Greene made a few remarks upon the treatment of fracture of the patella, and described a simple device which he had lately employed obtaining bony union. He places his patient on his back and puts the injured limb upon a single inclined plane, then straps on with adhesive plaster a piece of sole leather about two inches long and one wide, cut in the form of a crescent so that their concave surfaces will fit closely one to the superior and the other the inferior edges of the patella. After they have been firmly secured by adhesive strips he is able by further strapping to draw the fragments into close proximity. In one of the two cases reported there was considerable suppuration before the treatment was begun.

Dr. Cronyn said that when inflammatory action was very great there was more apt to be bony union, than without it. Although the leather supports might be new, the principle was the same as that embodied in the canvas knee cap, which consists of similar supports on opposite sides of the patella, which can be closely approximated by lacing after the cap has been made fast to the leg.

Dr. Rochester thought the apparatus an ingenious one, and that it might be made very useful in the treatment of this fracture. A firmer attachment could be obtained with adhesive plaster than with straps buckled around the leg. The straps and buckles caused irritation and sometimes sloughing, which was the principal objection to the knee cap; this, he thought could be obviated by using adhesive plaster in the manner suggested by Dr. Greene.

The next regular meeting night of the Association occurring upon the same date the American Medical Association convenes in this city, it was therefore moved and carried that the next regular meeting be held upon the evening of May 31st, to which date the Association then adjourned.

JOSEPH FOWLER, *Secretary.*

